# Rare case of gallbladder mucocele causing gastric outlet obstruction treated with cholecystectomy

**DOI:** 10.1016/j.ijscr.2019.03.013

**Published:** 2019-03-21

**Authors:** Wei-Liang Loh, Nick Zhi Peng Ng, Tousif Kabir, Chung Yip Chan

**Affiliations:** Department of Hepato-Pancreato-Biliary and Transplant Surgery, Academia Level 5, 20 College Road, Singapore, 169856, Singapore

**Keywords:** GOO, gastric outlet obstruction, CT, computer-tomography, CBD, common bile duct, MRCP, magnetic resonance cholangiopancreatography, Case report, Gallbladder mucocele, Gastric outlet obstruction, Cholecystectomy

## Abstract

**Introduction:**

Gallstone(s) impacted at the distal small bowel causing intestinal obstruction as a result of cholecystoenteric fistula is a well-known, albeit uncommon surgical condition. The rare Bouveret’s Syndrome, which refers to the proximal impaction of gallstone(s) in the duodenum or pylorus resulting in the gastric outlet obstruction (GOO), has also been described in the literature. However, gallbladder mucocele with extrinsic compression of the duodenum and/or pylorus causing GOO is a separate entity that is extremely rare.

**Presentation of case:**

A patient who presented with loss of appetite and weight, with intermittent vomiting over a course of eight months was found to have GOO secondary to extrinsic duodenal compression from a large gallbladder mucocele. Surgical intervention in the form of cholecystectomy was performed, and the patient’s symptoms resolved post-operatively.

**Discussion:**

We describe an extremely rare case of GOO, which can be easily corrected with cholecystectomy. Removal of the gallbladder removes the root cause of the issue, and should be first line therapy unless the patient is unfit for surgery.

**Conclusion:**

This extremely rare cause of GOO should be recognized, and treated promptly with surgery if possible.

## Highlights

• Gallbladder mucocele extremely rare etiology of GOO.
• Atypical symptoms made diagnosis difficult.
• Surgical intervention is suggested.

## 1 Introduction

Gallstones leading to enteric obstruction are a well-known entity. Gallstone ileus is an infrequent complication of cholelithiasis, occurring in less than 0.5 percent of patients with mechanical bowel obstruction and affecting females and the elderly disproportionately [1,2]. Bouveret’s Syndrome, is a distinct entity in which gastric outlet obstruction (GOO) develops after impaction of a large gallstone in the duodenum or pyloric channel usually secondary to a cholecystoenteric fistula [3]. However, iatrogenic cases of both conditions have been reported following laparoscopic cholecystectomy and endoscopic retrograde cholangiopancreatography (ERCP) with sphincterotomy [[4], [5], [6]].

The implicated gallstones usually are large enough in size, with the majority of stones larger than 2.5 cm in diameter [7]. Up to 70 percent of gallstones get impacted in the ileum, and the jejenum and gastric outlet are the next most frequently affected sites [2]. The colon is rarely the site of obstruction but can occur in the setting of diseased and strictured large bowel secondary to diverticular inflammation or inflammatory bowel disease [8,9].

An extremely rare cause of mechanical bowel obstruction secondary to cholelithiasis is that of a gallbladder mucocele, in which the size and position of the mucocele compresses on the duodenum/pylorus, causing GOO. This condition has only been described once before in the literature to the best of our knowledge [10].

We herein describe our experience with the second known case of this condition, which was treated definitively with cholecystectomy.

This work has been reported in line with the SCARE criteria [11].

## 2 Case report

The patient in question is a 63-year-old Chinese gentleman with a good performance status, a non-drinker and ex-smoker of seven pack years. He was diagnosed with Child-Pugh B8 liver cirrhosis secondary to chronic Hepatitis B infection in April 2017 after being admitted with liver decompensation with ascites.

During that admission, a work-up for symptomatic iron-deficiency anemia (hemoglobin 5.0 g/dL) revealed hypertensive gastropathy and four column Grade II–III esophageal varices on upper endoscopy, which were banded uneventfully. A colonoscopy revealed colonic edema indicative of portal hypertension. A triphasic computer-tomography (CT) scan of the liver showed irregular nodular contour of the liver with left lobe hypertrophy indicative of cirrhosis, with splenic and gastric cardia varices, splenorenal shunt, and severe ascites. The gallbladder was noted to be markedly distended then, but with no evidence of biliary or duodenal/pyloric obstruction. Ascitic fluid was sent for microbiology, which returned negative. He was started on medical therapy during that admission with improvement of his ascites and was discharged well.

A repeat CT scan in August 2017 showed a grossly dilated gallbladder, which was not seen to be compressing on the common bile duct (CBD) with mild prominence of the central intrahepatic biliary tree. The proximal small bowel loops were also noted to be mildly prominent with increased wall enhancement, likely related to portal hypertension.

**Fig. 1:**
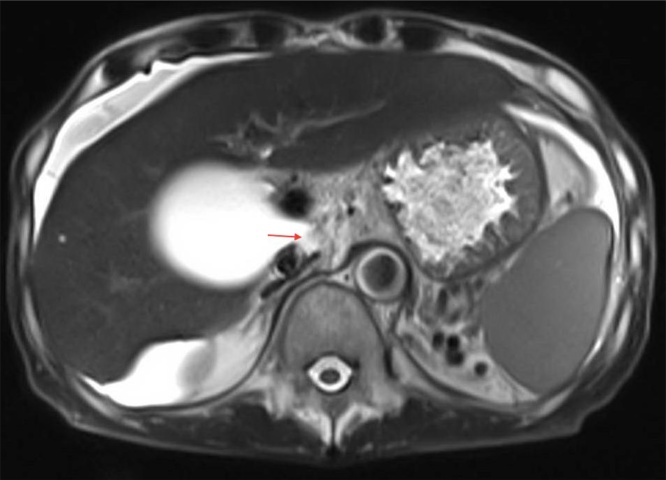
T2-weighted image demonstrating a hypointense focus in the expected locations of valves of Heister, depicted by a red arrow.

Given the concerning findings of gallbladder distension, an outpatient magnetic resonance cholangiopancreatography (MRCP) was performed. The MRCP performed in November 2017 showed a markedly distended, thin-walled gallbladder with a 1 cm gallstone lodged in the proximal cystic duct (Fig. 1). The CBD was stretched over the distended gallbladder with focal narrowing noted at its upper third related to the mass effect, with mild intrahepatic proximal dilation. The presence of loculated ascites, clustering of small bowel loops, and mild dilation of the proximal small bowel and duodenum raised the possibility of encapsulating peritonitis. The differential diagnoses for the patient at that point were intermittent GOO secondary to gallbladder mucocele, intermittent small bowel obstruction secondary to encapsulating peritonitis, and gastritis.

Cholecystectomy was offered to the patient with the aim of removing the potential source of GOO, with the caveat that his vomiting symptoms may not be fully resolved given the presence of other synchronous diagnoses. The patient declined surgical intervention at that point. The patient was admitted for postural hypotension likely secondary to intermittent vomiting in early March 2018, and a repeat CT scan once again demonstrated the distended gallbladder, with spontaneous resolution of his symptoms.

**Fig. 2:**
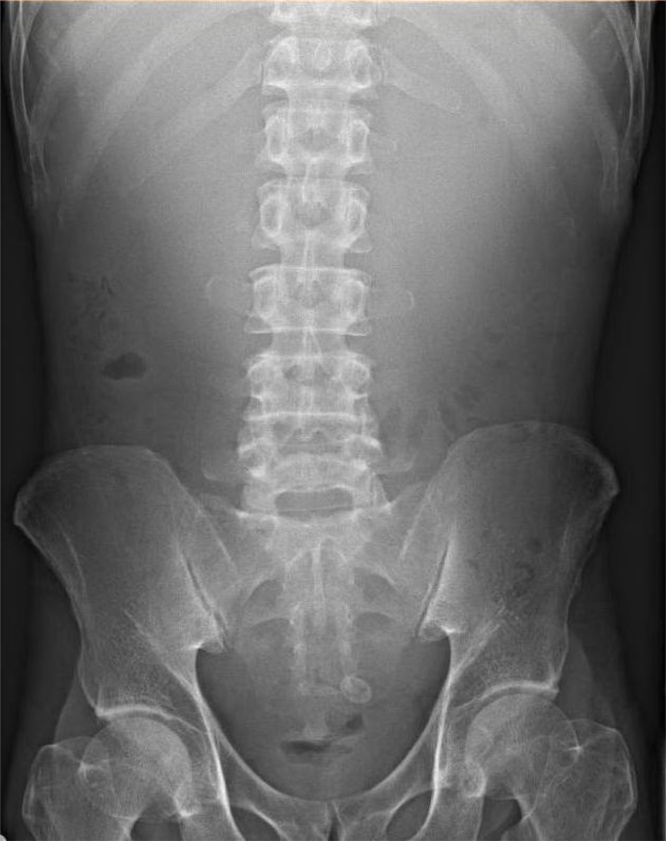
Paucity of bowel gas in the upper abdomen, with suggestion of a large mass (distended stomach) displacing bowel loops inferiorly).

**Fig. 3:**
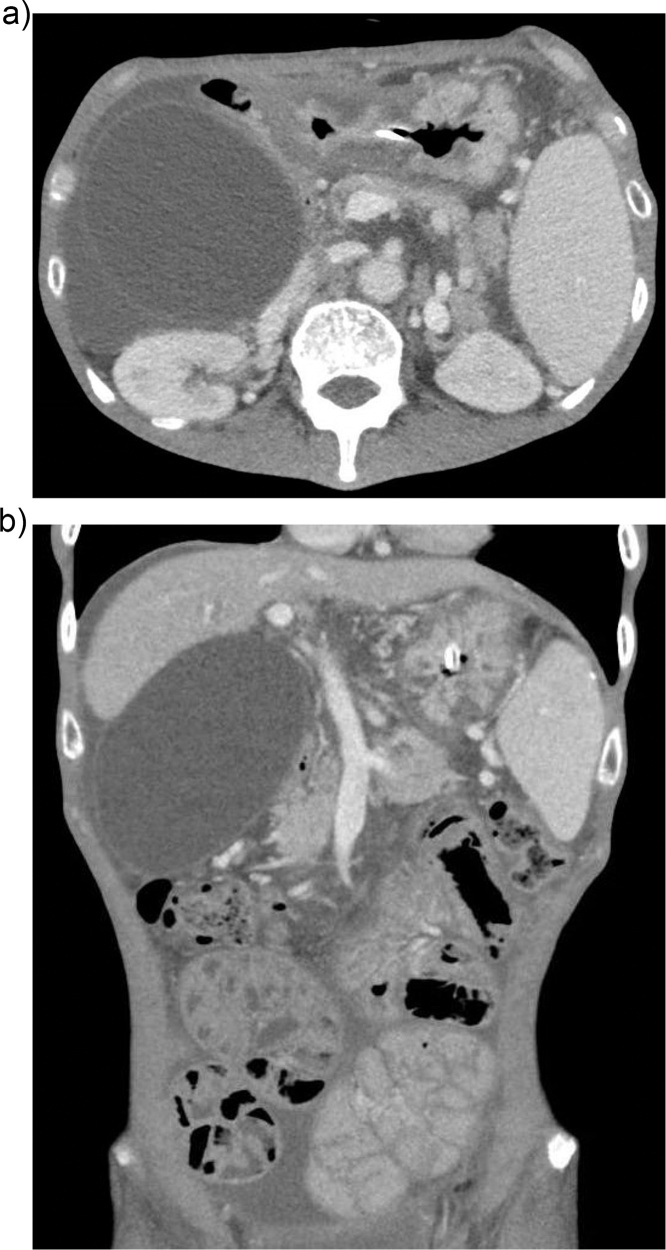
(a) Markedly distended gallbladder with ascites and compression of the duodenum. (b) Coronal section demonstrating the gallbladder mucocele and compression of the duodenum. Encapsulated small bowel loops with ascites can be appreciated in the left lower abdomen. The decompressed stomach with a nasogastric in-situ is visible in the upper abdomen.

His next admission in mid-April 2018 was the decisive one. He presented this time with markedly different symptoms; multiple episodes of non-bilious emesis and being unable to retain neither solids nor liquids. Abdominal examination revealed epigastric fullness with a succussion splash (Fig. 2). A nasogastric tube placed on intermittent suction removed three liters of yellowish effluent over the course of two days. By the time of a repeat CT abdomen pelvis two days after his admission, the output from the nasogastric tube had decreased significantly and no epigastric fullness nor succussion splash were clinically evident. Nevertheless, the CT showed a markedly distended gallbladder that had increased marginally in size, measuring 9.8 x 8.5 cm, with a resultant mass effect and compression on the second part of the duodenum. The small bowel loops were normal in caliber, but were unchanged in appearance and distribution compared to his last CT scan in March 2018 (Fig. 3a and b). It was determined that given the nature of symptoms and lack of small bowel dilation, that his concomitant encapsulating peritonitis was a red herring, and that the patient’s symptoms were due to GOO resulting from duodenal compression.

**Fig. 4:**
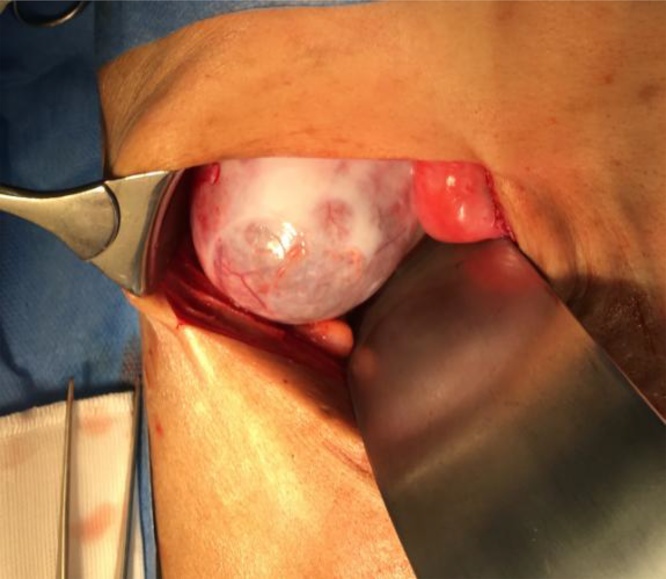
The patient’s head is towards the top of the image. A right subcostal incision has been made and the abdomen entered. The distended gallbladder mucoele with firm white fibrous plaques can be appreciated coming into view with retraction of the small bowel loops inferiorly.

An attempt was made for laparoscopic cholecystectomy despite the pre-operative expectation that it would likely not be possible in view of the encapsulating peritonitis. This proved to be true as safe entry could not be achieved via the umbilicus due to dense peritoneal adhesions, and the decision was made promptly for a right subcostal incision and open cholecystectomy. The distended gallbladder was covered with firm white plaques, and a cholecystostomy was performed to allow decompression of the gallbladder with a suction apparatus to enable surgical manipulation of the gallbladder and better visualization of biliary anatomy (Fig. 4). White mucoid bile was aspirated. A fundus first approach was used as the large size of the gallbladder even after decompression meant that the hepatocystic triangle could not be accessed immediately. A single pigmented stone 1 cm gallstone was found impacted at Hartmann’s pouch and was milked back into the gallbladder before securing and transecting the cystic duct. The small bowel loops were encapsulated within a firm white capsule but were not dilated, and the liver surface was similarly covered in the same white fibrous capsule; a biopsy of the peritoneal lining was taken for histological confirmation.

The patient recovered well, and was able to be taken off his nasogastric tube and tolerate diet by the second post-operative day, and was discharged by the third post-operative day. He was seen in clinic two weeks later with full resolution of his symptoms. The histology of the gallbladder showed features consistent with a gallbladder mucocele, and the peritoneal biopsy showed fibrous tissue with chronic inflammation and reactive mesothelial hyperplasia with no features of granulomatous inflammation or malignancy.

## 3 Discussion

This case highlights an extremely rare cause of GOO secondary to a gallbladder mucocele. A mucocele is defined by the distension of the gallbladder by an inappropriate accumulation of mucus. It is part of a complex disease process involving mild inflammation of the gallbladder wall with changes to its secretions, with decreased bile flow and gallbladder motility, and altered absorption of water from the lumen playing a part in the disease process. The largest reported gallbladder mucocele in the literature is 30 cm, in which surgeons successfully performed a laparoscopic cholecystectomy [12].

This patient also had Child-Pugh’s B liver cirrhosis. Morphological changes to the gallbladder associated with cirrhosis have been described in the literature, such as gallbladder varices in connection portal hypertension and portal vein thrombosis, and gallbladder wall thickening (congestive cholecystopathy) [[13], [14], [15]]. Whilst there has been no evidence thus far to suggest that cirrhosis and portal hypertension play a role in the development of gallbladder mucoceles, the theoretical possibility of alterations of gallbladder blood flow and secretions predisposing to mucoceles is worthy of investigation.

Interestingly, the diagnosis of GOO in this patient was delayed due to the coexistent presence of encapsulating peritonitis, which presumably was a result of his cirrhosis. His intermittency of symptoms, with episodes of vomiting followed by weeks of symptom relief exhibited that the GOO was initially irregular, and perhaps precipitated by positional changes and fluctuations in gallbladder size. It was only in the CT scan prior to surgery in which compression of the duodenum was convincingly demonstrated, thereby clinching the rare diagnosis.

The only other case of this condition reported was treated with percutaneous cholecystotomy with clinical resolution of symptoms. However, in our opinion, that allows an unacceptable high risk of recurrence, as the root cause of a gallstone impacted at the cystic duct/Hartmann’s pouch has not been dealt with. The definitive treatment is cholecystectomy, performed either laparoscopically or open depending on local expertise or technical or anatomical challenges, and should be offered to all surgically fit patients, with percutaneous cholecystotomy only performed in patients unfit for general anesthesia, or as a bridge to surgery.

## Conflicts of interest

None to declare.

## Sources of funding

None to declare.

## Ethical approval

This study is exempt from ethical approval in the authors’ institution.

## Consent

Consent was taken from the patient involved. The patient in question has been de-identified. A copy of the written consent is available upon request.

## Author’s contribution

Loh Wei-Liang: Conceptualising and writing of the paper

Nick Ng Zhi Peng: Assistant to writing of the manuscript and involved in care of patient.

Tousif Kabir: Collection of data and editing of manuscript

Chan Chung Yip: Main surgeon involved in care of patient and final editor of manuscript.

## Registration of research studies

researchregistry4281.

## Guarantor

Chan Chung Yip.

## Provenance and peer review

Not commissioned, externally peer-reviewed.
